# Supplementary material for: MetaRibo-Seq measures translation in microbiomes
Source: Nat Commun. 2020 Jun 29;11:3268. doi: 10.1038/s41467-020-17081-z (PMC7324362; doi:10.1038/s41467-020-17081-z)
Supplement: Supplementary file 10 — Supplementary Data 7 [file 41467_2020_17081_MOESM10_ESM.zip › File2/Confidence_High_Taxonomy/107896_out.krona.html]

Javascript must be enabled to view this page.

members
magnitude
magnitudeUnassigned
count
unassigned
taxon
rank

107896\_out

45

superkingdom
2
45

phylum
45
1239

186801
45
class

order
186802
45

family
186803
45

45

SRS042910\_contig\_number\_contig-100\_9366.65530
1
genus
1164882

467210
35
species

SRS011115\_contig\_number\_47534SRS011306\_contig\_number\_29013SRS014573\_contig\_number\_contig-100\_2148.181783SRS015797\_contig\_number\_contig-100\_139.169569SRS016037\_contig\_number\_25697SRS016086\_contig\_number\_13226SRS016319\_contig\_number\_contig-100\_17079.48006.48006SRS018145\_contig\_number\_contig-100\_6064.101158SRS018679\_contig\_number\_14694SRS018739\_contig\_number\_contig-100\_10135.10135.10135SRS019327\_contig\_number\_contig-100\_3115.191975.191975SRS019607\_contig\_number\_22708SRS022530\_contig\_number\_contig-100\_2428.87764.87764SRS023617\_contig\_number\_10462SRS023835\_contig\_number\_contig-100\_14496.45027SRS024015\_contig\_number\_5039SRS047219\_contig\_number\_contig-100\_3652.126932SRS049389\_contig\_number\_49699SRS054079\_contig\_number\_11214SRS063193\_contig\_number\_30825SRS063288\_contig\_number\_contig-100\_2542.71537SRS065099\_contig\_number\_contig-100\_3730.3730SRS075404\_contig\_number\_contig-100\_934.210365SRS075987\_contig\_number\_35468SRS076951\_contig\_number\_contig-100\_385.234917SRS077520\_contig\_number\_contig-100\_2661.435305SRS077568\_contig\_number\_21214SRS098867\_contig\_number\_contig-100\_6340.35391SRS104519\_contig\_number\_17659SRS142896\_contig\_number\_contig-100\_4750.156800SRS143423\_contig\_number\_34409SRS143529\_contig\_number\_contig-100\_4977.359122SRS146996\_contig\_number\_contig-100\_432.153029SRS149486\_contig\_number\_contig-100\_3061.109235SRS149961\_contig\_number\_9835


SRS014271\_contig\_number\_contig-100\_1622.198189SRS057931\_contig\_number\_contig-100\_4187.65511SRS077300\_contig\_number\_contig-100\_9127.188191SRS098045\_contig\_number\_contig-100\_3401.94181
species
936595
4

species
979627
5

SRS014935\_contig\_number\_17074SRS021496\_contig\_number\_38311SRS062544\_contig\_number\_3851SRS075947\_contig\_number\_33303SRS142787\_contig\_number\_47571
